# Discovery of temporal and disease association patterns in condition-specific hospital utilization rates

**DOI:** 10.1371/journal.pone.0172049

**Published:** 2017-03-29

**Authors:** Julian S. Haimovich, Arjun K. Venkatesh, Abbas Shojaee, Andreas Coppi, Frederick Warner, Shu-Xia Li, Harlan M. Krumholz

**Affiliations:** 1 Albert Einstein College of Medicine, Bronx, New York, United States of America; 2 Center for Outcomes Research and Evaluation, Yale-New Haven Hospital, New Haven, Connecticut, United States of America; 3 Department of Emergency Medicine, Yale University School of Medicine, New Haven, Connecticut, United States of America; 4 Section of Cardiovascular Medicine, Department of Internal Medicine, Yale School of Medicine, New Haven, Connecticut, United States of America; 5 The Robert Wood Johnson Foundation Clinical Scholars Program, Department of Internal Medicine, Yale School of Medicine, New Haven, Connecticut, United States of America; 6 Department of Health Policy and Management, Yale School of Public Health, New Haven, Connecticut, United States of America; Columbia University, UNITED STATES

## Abstract

Identifying temporal variation in hospitalization rates may provide insights about disease patterns and thereby inform research, policy, and clinical care. However, the majority of medical conditions have not been studied for their potential seasonal variation. The objective of this study was to apply a data-driven approach to characterize temporal variation in condition-specific hospitalizations. Using a dataset of 34 million inpatient discharges gathered from hospitals in New York State from 2008–2011, we grouped all discharges into 263 clinical conditions based on the principal discharge diagnosis using Clinical Classification Software in order to mitigate the limitation that administrative claims data reflect clinical conditions to varying specificity. After applying Seasonal-Trend Decomposition by LOESS, we estimated the periodicity of the seasonal component using spectral analysis and applied harmonic regression to calculate the amplitude and phase of the condition’s seasonal utilization pattern. We also introduced four new indices of temporal variation: mean oscillation width, seasonal coefficient, trend coefficient, and linearity of the trend. Finally, K-means clustering was used to group conditions across these four indices to identify common temporal variation patterns. Of all 263 clinical conditions considered, 164 demonstrated statistically significant seasonality. Notably, we identified conditions for which seasonal variation has not been previously described such as ovarian cancer, tuberculosis, and schizophrenia. Clustering analysis yielded three distinct groups of conditions based on multiple measures of seasonal variation. Our study was limited to New York State and results may not directly apply to other regions with distinct climates and health burden. A substantial proportion of medical conditions, larger than previously described, exhibit seasonal variation in hospital utilization. Moreover, the application of clustering tools yields groups of clinically heterogeneous conditions with similar seasonal phenotypes. Further investigation is necessary to uncover common etiologies underlying these shared seasonal phenotypes.

## Introduction

Epidemiological studies have identified temporal variation in hospitalization rates across different conditions, particularly with respect to seasons. These studies have shown significant temporal variation with periodicity ranging from day of the week to season for specific diseases varying in etiological origin and health burden. Significant seasonal temporal variation of hospital utilization rates has been shown for acute diseases such as stroke [1], venous thromboembolism [2, 3], influenza [4], acute myocardial infarction [5], as well as for chronic diseases including chronic obstructive pulmonary disease [6], heart failure [7, 8], atrial fibrillation [9], and psychiatric disorders [10] such as bipolar disorder and mood disorders [11]. Interestingly, each of these previously studied diseases exhibits annual utilization peaks in the winter.

However, there remain several gaps in knowledge. Previous work studying temporal patterns in hospital utilization rates has largely applied traditional statistical approaches that begin with a hypothesis regarding variation of a single disease driven by previously identified seasonal mediators. These mediators range from infectious and microbiologic triggers [12] to environmental factors such as temperature [13] and pollution [14]. Such traditional approaches are unlikely to uncover unexpected temporal patterns that may be revealed only by broad data mining techniques applied across many conditions. While one previous study utilized a data mining approach, it was limited to the analysis of only one-fifth of diseases with the highest admission rates at a single institution, leaving the majority of diseases across a broader population unstudied [15]. In addition, past studies have reduced the complexity of temporal pattern analysis to three simple indices, namely amplitude, period, and phase, which together provide only a limited characterization of temporal variation.

Accordingly, we sought to characterize temporal variation in hospital utilization rates for all available clinical conditions, and use these patterns to identify underlying similarities amongst conditions. We applied a data-driven approach to study seasonal variation in hospital utilization among all clinical conditions using a statewide, all-payer, longitudinal dataset of inpatient hospitalizations and emergency department visits. To characterize the temporal patterns embedded in the data, we developed an algorithm that combines the extraction of traditional indices of amplitude, period, and phase, with four newer indices of temporal variation: seasonal coefficient, mean oscillation width, trend coefficient, and trend linearity. The addition of these indices, the value of which has been demonstrated in fields such as environmental science [16], enabled a more detailed description of temporal variation in individual diseases and the application of clustering tools to identify groupings of diseases based on their multi-dimensional characterization of temporal variation in hospitalizations.

## Materials and methods

### Study design

This study was approved by the Yale School of Medicine Institutional Review Board with a waiver of consent. We employed the 2008–2011 New York State Inpatient Databases (SID) [17] and State Emergency Department Databases (SEDD) [18] encompassing nearly 40 million records. The SID and SEDD are made available through the Agency for Healthcare Research and Quality’s (AHRQ) Healthcare Cost and Utilization Project, and include administrative claims data submitted by all participating hospitals in New York State. The Healthcare Cost and Utilization Project datasets contain encounter-level information, including all-listed diagnoses (as recorded at time of discharge), discharge status, patient demographics, and the month and year of inpatient admission in the case of SID, and visit in the case of SEDD. We define utilization for a given condition as the initiation of hospital care through either emergency department visits, or inpatient care with the condition of interest recorded as the principal discharge diagnosis. We combined these outcomes to capture the totality of acute care utilization.

### Subjects

We included all patients discharged from an inpatient hospital stay or from a treat-and-release emergency department visit. Stays or visits that resulted in in-hospital death were also included. To facilitate condition-specific analyses, we grouped all discharges based on the International Classification of Diseases, Ninth Revision, Clinical Modification (ICD-9-CM) principal discharge diagnosis using AHRQ’s Clinical Classification Software (CCS). The CCS organizes more than 14,000 ICD-9-CM codes into 263 common, meaningful clinical condition categories [19]. Of the original 263 codes, 246 (94%) were included in our analysis. Exclusions are summarized in S1 Fig.

### Data pre-processing

In order to ensure data quality, we implemented multiple processing steps to exclude data points with year or state discrepancies relative to the dataset publisher’s specified information. Following exclusion of such data points, we subsequently included data only with appropriately labeled month (e.g. is labeled and numerically corresponds to a month of the year) and the diagnosis code of interest.

### Time series methods

To evaluate seasonal variation in condition-specific hospital utilization rates, we conducted a series of analyses that first applied seasonal-trend decomposition to extract the seasonal component of the time series. The seasonal component is a high frequency repeating pattern present in the data. Following seasonal-trend decomposition, we applied harmonic regression and spectral analysis to extract three key indices characterizing seasonal variation: period, amplitude, and acrophase. We then calculated an additional four indices of seasonal variation: seasonal coefficient, mean oscillation width, trend coefficient, and trend linearity. Finally, we applied k-means clustering to group conditions with statistically significant (yearly) seasonality based on these four additional indices of temporal variation, which also demonstrate independence. Eight selected examples of raw utilization data are shown in S2 Fig.

### Seasonal-trend decomposition based on LOESS

**Fig 1:**
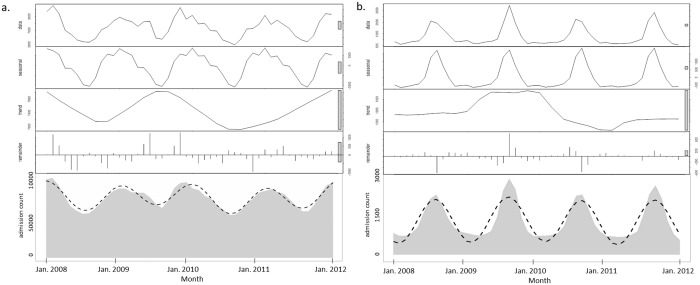
STL decomposition for monthly condition-specific hospitalization rates. (a.) Pneumonia (CCS 122) peaks in the winter. (b.) Poisoning by non-medicinal substances (CCS 243) peaks in the summer. Horizontal axis: time of hospital utilization. Top panel: original time series. For both the seasonal component and residual component, the vertical axis is the variation in admissions; for the trend component, the axis represents a count of admissions. The shaded bar shown on the right is the degree by which the plot must be shrunk to display the component on the same scale as the raw data. A larger bar indicates that a greater amount of shrinking is required, and therefore, that variations in a component with a large bar are small relative to variations in the data. Bottom panel: harmonic regression model fit of seasonal component summed with trend component (dashed black line) plotted over original time series.

After grouping all hospitalizations in the dataset by principal CCS diagnosis, we constructed a condition-specific time series according to month and year of hospital utilization. The Seasonal-Trend Decomposition based on LOESS (STL), where “LOESS” stands for local regression, was then applied to decompose each condition-specific hospital utilization time series into trend, seasonal, and remainder components [20]. STL assumes that the underlying data can be represented by the sum of the trend, seasonal, and remainder components. The STL algorithm first identifies the trend component, and subsequently calculates the seasonal component following removal of this trend component. Past studies have shown the importance of de-trending time series prior to analyzing temporal variation [21]. Following de-trending, the seasonal component was calculated by local linear regression. This component represents a periodical variation of the time series. We then applied spectral analysis to determine the periodicity of the seasonal component, and harmonic regression to obtain a sinusoidal approximation of the seasonal component. The remainder component, the result of removing both the seasonal and trend components from the time series, was not used in the analysis. Fig 1 demonstrates two examples of STL analysis and harmonic regression for the monthly condition-specific utilization rates of pneumonia (CCS 112), a condition for which utilization rate peaks in the winter (Fig 1a), and poisoning by non-medicinal substances (CCS 243), a condition for which utilization rate peaks in the summer (Fig 1b).

### Spectral analysis and harmonic regression

**Fig 2:**
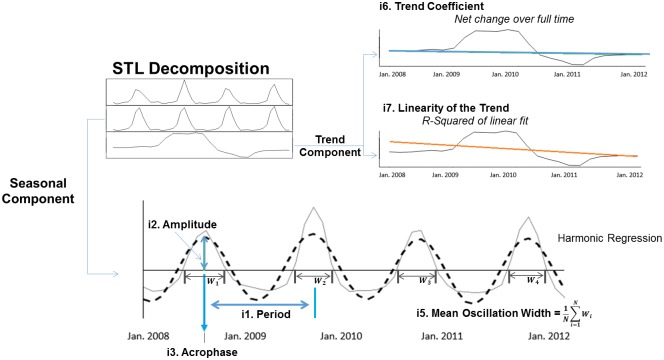
Analytical approach and calculation of indices of temporal variation. STL analysis is first carried out on the raw hospital utilization time series in order to separate the data into the seasonal and trend components. Harmonic regression is then used to fit a sinusoidal curve (dashed black line) to the seasonal component of the data in order to measure the period (i1), amplitude (i2), and phase. These indices are used to determine the seasonality of the data, as well as to quantify other indices including the acrophase (i3) and seasonal coefficient (i4). The mean oscillation width is calculated from the seasonal component directly (i5). In addition to analysis of the seasonal component, a linear fit to the trend component is used to determine the trend coefficient (i6) and linearity of the trend (i7).

We applied periodogram spectral analysis with subsequent harmonic regression to calculate three indices of temporal variation that have been well-described: period, amplitude, and acrophase. Fig 2 provides an example of how these values, together with new indices of temporal variation described below, were calculated. We first applied periodogram spectral analysis to the seasonal component of the data to determine the dominant periodicity (i1) present in the data. We then smoothed the data using a 3-sample moving average and applied harmonic regression to the seasonal component of the data to extract two indices of seasonality that have been well-described previously: (i2) amplitude and (i3) acrophase.

### New indices of temporal variation

Along with the traditional indices of temporal variation obtained from harmonic regression (period, amplitude, and acrophase), our analysis incorporated four new indices characterizing seasonality (i4-i7): the seasonal coefficient, mean oscillation width, trend coefficient, and the linearity of trend. These additional indices reflect a new application of established numerical methods to further describe properties of temporal variation in condition-specific hospital utilization time series. As explained below in more detail, the seasonal coefficient (i4) captures the relative change in utilization rate due to season, the mean oscillation width (i5) reflects temporal properties of the seasonal component of the time series, while the trend coefficient (i6) and linearity of the trend (i7) reflect temporal properties of the trend component. Fig 2 demonstrates how these indices are calculated using CCS 243 as an example.

**Time-series indices definitions:**
i1 Period: the time interval between oscillatory peaks of the seasonal component. A period of 12 months suggests that the pattern of utilization repeats itself every 12 months.
i2 Amplitude: the amplitude was calculated as a measure of the peak oscillatory height of the sinusoidal model fit to the seasonal component by harmonic regression. The amplitude describes the magnitude of hospital utilization variation over one period—the larger the amplitude, the greater the seasonal effect on the number of utilizations over one period.
i3 Acrophase: the time at which the first peak of the sinusoidal model fit to the seasonal component occurs. Although the acrophase is defined via the first peak, since the data are sinusoidal, subsequent peaks occur at times corresponding to the acrophase plus integer multiples of the period. Therefore, if the data have yearly variation, the acrophase can be more broadly interpreted as the time of the year when utilization peaks.
i4 Seasonal coefficient: the ratio of amplitude to the mean monthly utilization rate of the raw utilization data. This value can be used to interpret the effect of seasonality on peak utilization rate (amplitude) in relation to the average, baseline utilization rate of a given condition. The larger the seasonal coefficient, the more pronounced the seasonal effect is on utilization rate relative to the mean monthly utilization rate.
i5 Mean oscillation width: the average length of the time interval during which oscillations of the seasonal component are greater than zero. This value can also be interpreted as the duration of the seasonal effect on monthly utilizations. A smaller mean oscillation width suggests that during the season of peak utilization, as determined by acrophase, there is a sharper rise and fall in utilizations. A larger width would suggest that there is a more prolonged increase in utilization rates during the season of peak admission for a given condition.
i6 Trend coefficient: the ratio of the mean of first order differences in the values of the trend (trend velocity) to the mean monthly utilization rate of the raw utilization data. The trend coefficient is a measure of the broad change in utilization rate over years in relation to the baseline utilization rate of the condition. The sign of the trend coefficient determines if the utilization rate is increasing or decreasing over time, and the magnitude of the trend coefficient represents the size of those changes relative to baseline utilization rates.
i7 The linearity of the trend: the linear characteristic of the trend is determined by the r-squared value of a linear fit of the trend line. The higher the r-square, which has a maximum value of 1, the more linear the trend.

### Clustering analysis

After identifying clinical conditions with statistically significant seasonal variation, we sought to utilize both traditional and new indices of temporal variation to identify clusters of conditions with similar temporal patterns. Only conditions with a period equal to 12 months and a statistically significant F-test were considered seasonal and included in the clustering analysis. Seasonal conditions were stratified based on acrophase, which quantifies the time of year when the utilizations for a given condition peak. K-means clustering was then applied to cluster diseases that had peak utilization rates only in the summer time (June, July, and August), which encompassed the majority of diseases.

K-means clustering is an unsupervised clustering algorithm that partitions the multidimensional dataset of conditions and their respective indices into a set number (k) of clusters indices. K-means clustering was chosen among other clustering methods due to its widespread usage and well-defined and easily interpretable clustering quality measures. Clustering was based on four indices of temporal variation: seasonal coefficient, mean oscillation width, trend coefficient, and trend linearity. Independence of these indices prior to clustering was demonstrated using the Pearson correlation coefficient. Data were normalized and the Euclidean similarity measure was used. Period and acrophase were not included in clustering because they were used as a criterion for inclusion in clustering. Amplitude was also excluded because it was used to calculate the seasonal coefficient.

Clustering performance was evaluated by calculating the average silhouette width which captures the average difference between the distance a point has to the center of the cluster into which it was grouped and the distance between that point and the center of the nearest neighboring cluster [22]. Specifically, we calculated the silhouette width for the number of clusters (k) ranging from 2 to 10, repeating the clustering procedure 200 times for each value (k), and calculating the silhouette width for every iteration. The 3-cluster solution had the best performance (Si = 0.47) and was therefore selected as the optimal clustering solution.

Clustering visualization utilizes discriminant coordinates to reduce the dimensionality of the dataset to a two-dimensional representation [23]. The two coordinate axes for cluster display are chosen as the vector maximizing the ratio of the between group variance to the within group variance. Datasets are then projected onto these coordinates to generate the cluster plot.

### Software packages and parameter selection

Quantitative analysis was done using R version 3.2.0 [24]. Seasonal decomposition of time series by LOESS utilized the STL function with parameters s.window = 7 (span of LOESS window used for seasonal extraction), s.degree = 0 (degree of locally-fitted polynomial in seasonal extraction), and t.window = 19 (span of LOESS window used for trend extraction). The built-in spectrum function was used to determine the dominant periodicity of the seasonal component. Harmonic regression analysis relied upon the open source ‘HarmonicRegression’ package with the tau parameter set to the period estimated by spectral analysis. Pairwise Pearson correlation was calculated using the cor function with method “pearson”. K-means clustering utilized the default ‘kmeans’ package. Silhouette width was calculated using the cluster.stats function published as part of the open source ‘fpc’ package. Cluster plotting was carried out using the plotcluster function, another function published in the ‘fpc’ package.

### Statistical analysis

In order to characterize the temporal variation in the seasonal component of the data, and thereby determine whether the seasonal component has statistically significant seasonal variation, we applied the F-test. The F-test is a measure of the goodness-of-fit of the sinusoidal model to the seasonal component of a condition-specific hospital utilization time series. A statistically significant p-value (p < 0.05) suggests that the seasonal component is well-fit by a sine curve with period estimated via spectral analysis. Furthermore, if the period calculated by spectral analysis is 12 months, this means that the condition has a yearly pattern of hospital utilization that peaks repetitively at the same time each year.

In order to assess the impact of random (Gaussian) noise on the determination of statistically significant seasonality in these condition-specific utilization time series, 1000 random time series composed of samples drawn from a normal distribution were analyzed for seasonal variation. We then compared the proportion of conditions deemed to have statistically significant seasonality to the number of random samples deemed to have statistically significant seasonality, to verify that the latter is small.

## Results

### Summary of temporal variation in hospital utilization rates

During the years 2008–2011, of the 33.6 million hospital visits in New York State, 9.8 million were inpatient hospital stays. The annual inpatient admission rate and emergency department visit rate remained stable over the three years, at approximately 13,000 visits per 100,000 people and 30,000 visits per 100,000 people, respectively.

Spectral analysis yielded 164 of 246 conditions (66.7%) with a seasonal component with an estimated period of 12 months. Among these 164 conditions, all harmonic regression model fits of seasonal components were statistically significant (p-value < 0.05) indicating that all seasonal components of the condition-specific utilization time series demonstrated significant yearly variation. In contrast, of 1000 random samples, only 60 (6%) met criteria for statistically significant seasonality, indicating robustness of this analytical approach to noise.

**Fig 3:**
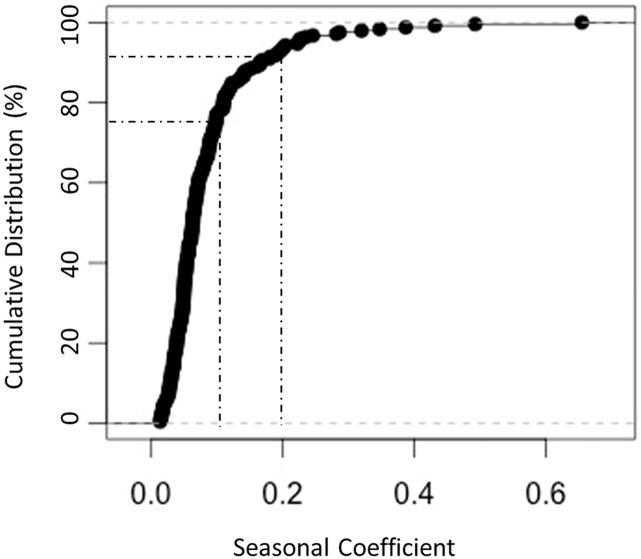
Cumulative distribution of seasonal coefficient for 164 conditions with statistically significant seasonal variation. Displays the percentage of diseases (y-axis) that have less than or equal to the specified seasonal coefficient (x-axis). Fifty codes (30.5%) have a seasonal coefficient greater than 0.1 and 15 codes (9.1%) have a seasonal coefficient greater than 0.2.

The cumulative distribution plot for the seasonal coefficient identified 15 (9.1%) conditions with a seasonal coefficient greater than 0.2 and 50 (30.5%) conditions with a seasonal coefficient greater than 0.1 (Fig 3). This indicates that for more than 30% of conditions with statistically significant seasonality, the peak change in hospital utilization was greater than or equal to 10% of the mean monthly utilization rate.

Stratification of conditions based on acrophase showed that the majority of conditions have peak utilization rates in the summer (78.6%), and peak utilization rates occur least often in the fall (4.8%). The other conditions peak in the winter (6.7%) and spring (9.8%). Tables 1 and 2 summarize these results, and also display conditions that have the top five amplitudes and seasonal coefficients, respectively.

**Table 1:** Highest amplitude conditions with seasonal variation in hospital utilization by season.

Season (n^a^)	Highest amplitude conditions	Amplitude	Seasonal coefficient
Winter (11)	Other upper respiratory infection (CCS: 126) Acute bronchitis (CCS: 125) Otitis media and related conditions (CCS: 92) Pneumonia (except that caused by tuberculosis or sexually transmitted disease) (CCS: 122) Nausea and vomiting (CCS: 250)	5996 2301 1925 1503 1209	0.23 0.43 0.25 0.20 0.23
Spring (16)	Noninfectious gastroenteritis (CCS: 154) Other upper respiratory disease (CCS: 134) Intestinal infections (CCS: 135) Fluid and electrolyte disorders (CCS: 55) Gastritis and duodenitis (CCS: 140)	2456 805 575 329 265	0.35 0.17 0.22 0.08 0.07
Summer (129)	Superficial injury; contusion (CCS: 239) Open wounds of extremities (CCS: 236) Skin and subcutaneous tissue infections (CS: 197) Sprains and strains (CCS: 232) Other injuries and conditions due to external causes (CCS: 244)	4949 4233 2655 2240 1891	0.18 0.29 0.20 0.09 0.11
Fall (8)	Intracranial injury (CCS: 233) Inflammatory diseases of female pelvic organs (CCS: 168) Other fractures (CCS: 231) Meningitis (CCS: 76) Non-malignant breast conditions (CCS: 167)	197 142 118 60 40	0.07 0.07 0.06 0.32 0.05

Amplitude, the peak value of the seasonal component and a measure of the seasonal impact on raw number of utilizations for a given condition; Seasonal coefficient, the amplitude divided by the mean utilization rate, which therefore is a measure that describes the effect of season on utilizations relative to the mean utilization rate for a given condition; CCS, Clinical Classification Software.

^a^ Number of conditions for each season is reported.

**Table 2:** Highest seasonal coefficient conditions with seasonal variation by season.

Season (n^a^)	Highest amplitude conditions	Seasonal coefficient	Amplitude
Winter (11)	Acute bronchitis (CCS: 125) Otitis media and related conditions (CCS: 92) Nausea and vomiting (CCS: 250) Other upper respiratory infection (CCS: 126) Pneumonia (except that caused by tuberculosis or sexually transmitted disease) (CCS: 122)	0.43 0.25 0.23 0.23 0.21	2301 1925 1209 5996 1503
Spring (16)	Bacterial infection; unspecified site (CCS: 3) Noninfectious gastroenteritis (CCS: 154) Intestinal infections (CCS: 135) Other upper respiratory disease (CCS: 134) Gangrene (CCS: 248)	0.39 0.35 0.22 0.17 0.13	116 2456 575 805 30
Summer (129)	Poisoning by nonmedicinal substances (CCS: 243) Open wounds of extremities (CCS: 236) Other infections; including parasitic (CCS: 8) Short gestation; low birth weight; and fetal growth retardation (CCS: 219) Allergic reactions (CCS: 253)	0.65 0.29 0.28 0.23 0.22	846 4233 242 7 1696
Fall (8)	Meningitis (CCS: 76) Disorders of lipid metabolism (CCS: 53) Cataract (CCS: 86) Other non-epithelial cancer of skin (CCS: 23) Intracranial injury (CCS: 233)	0.32 0.15 0.11 0.10 0.07	60 4 3 4 197

Amplitude, the peak value of the seasonal component and a measure of the seasonal impact on raw number of utilizations for a given condition; Seasonal coefficient, the amplitude divided by the mean utilization rate, which therefore is a measure that describes the effect of season on utilizations relative to the mean utilization rate for a given condition; CCS, Clinical Classification Software.

^a^ Number of conditions for each season is reported.

### Pairwise Pearson correlation of temporal indices used in clustering

**Fig 4:**
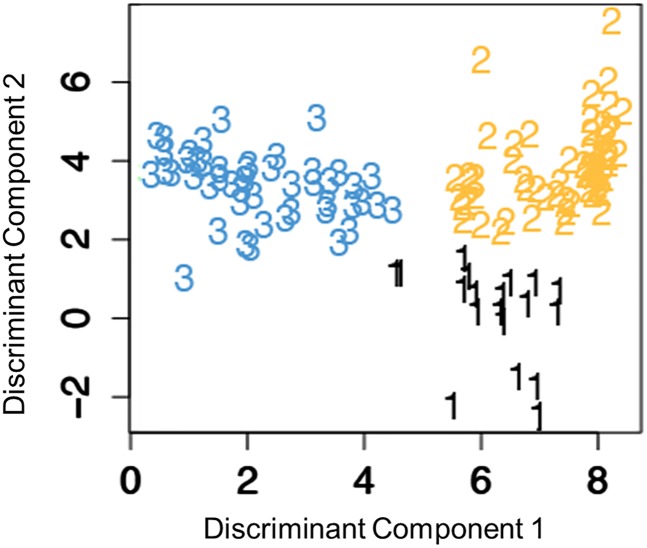
Clustering of 129 seasonal conditions with summer peaks plotted against the first two discriminant components. Clustering was based on four independent indices of temporal variation including mean seasonal coefficient, mean oscillation width, trend coefficient, and trend linearity as determined by quantitative analysis. The optimal cluster solution of k = 3 clusters was validated using the silhouette width (Si = 0.47) as a metric.

Pairwise Pearson correlation coefficients calculated amongst each of the four indices included in clustering analysis of conditions that peak in the summer were consistently lower than 0.4 as demonstrated in Table 3. This result suggests that the indices are not strongly correlated with each other, and that each index provides some unique and informative data. Fig 4 displays clustering for the optimal solution of k = 3 clusters using discriminant coordinates as described in the methods. Centroids are shown in Table 4 and represent the average of each index across all conditions in a given cluster.

**Table 3:** Pairwise Pearson correlation coefficients for indices of temporal variation for conditions with summer peaks included in k-means clustering.

Index	Seasonal coefficient	Mean oscillation width	Trend coefficient	R-square
Seasonal coefficient	-	-0.28 CI^a^: [-0.43, -0.11]	-0.12 CI^a^: [-0.28, 0.06]	-0.14 CI^a^: [-0.31, 0.03]
Mean oscillation width	-	-	-0.18 CI^a^: [-0.34, -0.01]	-0.05 CI^a^: [-0.22, 0.12]
Trend coefficient	-	-	-	0.35 CI^a^: [0.19, 0.50]

^a^ 95% CI of correlation coefficients are shown for each pair of indices.

**Table 4:** Centroids of 3-cluster k-means grouping of conditions with summer peaks in utilization.

Cluster	Number of conditions	Seasonal coefficient	Mean oscillation width	Trend coefficient	Trend linearity
1	49	0.10	6.38	0.52 × 10^−3^	0.24
2	61	0.08	6.24	3.95 × 10^−3^	0.88
3	19	0.10	6.60	-2.88 × 10^−3^	0.80

### Clustering of conditions based on characteristics of temporal variation

Cluster group 1 contains 49 conditions (38.0%) with the smallest trend coefficient and a relatively large seasonal coefficient. Cluster 1 therefore contains conditions that exhibit a pronounced increase in admission rate in the summer and relatively small, non-linear changes in utilization over time. This group includes traumatic injuries including fractures of lower limbs (CCS: 230); sprains and strains (CCS: 232); burns (CCS: 240); and poisoning by non-medicinal substances (CCS: 243); central nervous system diseases such as multiple sclerosis (CCS: 80); Parkinson’s disease (CCS: 79); and some cancers of the gastrointestinal tract including stomach (CCS: 13) and pancreatic (CCS: 17).

Cluster group 2 contains 61 conditions (47.3%) with the smallest seasonal coefficient, the smallest mean oscillation width, and the largest trend coefficient. Cluster group 2 therefore contains conditions that exhibit neither dramatic nor prolonged increases in utilization rates in the summer relative to conditions in other clusters. Though the effect of season on the utilization rates of these conditions is relatively small, their utilization is increasing in a linear pattern over time. This group includes psychiatric conditions including anxiety disorders, mood disorders, alcohol-related disorders, and substance-related disorders (CCS: 651, 653, 660, 661, respectively); allergic reactions (CCS: 253); and other sensory disorders such as other ear and sense organ disorders (CCS: 94) and blindness and vision defects (CCS: 89).

Cluster group 3 contains 19 conditions (14.7%) with the largest mean oscillation width and negative trend coefficients. Cluster group 3 therefore contains conditions that exhibit a pronounced and prolonged increase in utilizations during the summer. Furthermore, the utilization of these conditions is decreasing in a linear pattern over time. This group contains respiratory conditions such as respiratory distress syndrome (CCS: 221) and tuberculosis (CCS: 1); gynecological conditions including neoplasms of the cervix and uterus (CCS: 26, 46, respectively); and conditions related to birthing: fetopelvic disproportion and obstruction (CCS: 188), fetal distress and abnormal forces of labor (CCS: 190), malposition and malpresentation (CCS: 187), and cardiac and circulatory congenital anomalies (CCS: 213). S1 Table provides a comprehensive list of conditions and their respective clustering assignments.

## Discussion

Our analysis of condition-specific hospital utilization rates using a computational approach to characterize seasonal variation found that two-thirds of studied conditions demonstrate statistically significant seasonal hospital utilization. Additionally, using new indices enabled both a more precise characterization of temporal variation, as well as clustering of conditions based on their seasonal patterns, which may support future investigations seeking to understand the common triggers or circumstances amongst clinically heterogeneous conditions.

Consistent with prior work focusing on the study of temporal variation in single conditions, our analysis showed that conditions such as chronic obstructive pulmonary disease, venous thromboembolism, congestive heart failure, atrial fibrillation, and stroke have winter peaks in their utilization rates. While summary review of previous single disease studies may suggest that utilization rates tend to peak in the winter, our analysis of temporal variation in a diverse collection of clinical conditions has shown that summer peaks predominate. Furthermore, previous work has provided only a narrow understanding of temporal variation in hospital utilization whereas the largest study we identified was limited to only the top one-fifth of conditions with the largest mean monthly admission rate [15]. Here, we have dramatically expanded understanding of temporal variation through the study of 246 condition-specific hospital utilization rates, which together encompass nearly all medical conditions.

In addition, we have included new indices in order to more precisely describe temporal variation, and allow for the application of clustering tools. Our work extends the use of indices, like trend linearity, which have been commonly used in other disciplines from environmental science [25] and parasitology [26] to healthcare services. According to our analysis, traditional indices such as acrophase and period are highly similar amongst statistically significant conditions whereas by definition, all statistically significant conditions have a period of 12 months, and we have shown that nearly 80% of conditions peak in the summer. This homogeneity precludes the ability of clustering tools to identify meaningful groupings of conditions, whereas the new indices introduced here demonstrate independence and generate interesting centroids from clustering. Therefore, the introduction of new indices of temporal variation has not only expanded our understanding of temporal variation in individual conditions, but it has also laid the foundation for the discovery of new subgroups of conditions.

Our clustering analysis showed three clusters or seasonal phenotypes. Cluster group 1 contains a range of traumatic injuries that are likely to occur together during the summer, including poisoning by non-medicinal substances (such as alcohol) and sprains, strains, and fractures. These types of injuries may be those typically associated with vacations and recreation and known summer peaks in motor vehicle crashes [27]. Cluster 2, the largest cluster with approximately 47% of summer conditions, contains both substance use disorders and psychiatric conditions, which is consistent with previous findings, showing significant temporal connections between the development of alcohol and substance-related disorders and psychiatric disorders like anxiety, mood, and personality disorders [28]. Cluster 3, the smallest cluster with about 15% of summer conditions, contains conditions related to abnormal fetal positioning and other diagnoses related to complicated birthing [29]. More generally, clustering of conditions may suggest common temporal relationships among conditions not previously known to share clinical characteristics such as cancer of the ovary or liver and the many infectious conditions found in Cluster 2. The future study of seasonal triggers underlying temporal similarities among clinically different diseases has the potential to uncover previously unknown mechanisms of disease onset.

Our study has several limitations. First, our analyses were conducted using administrative claims data, which may reflect clinical conditions with varying specificity. However, our use of the CCS grouping, which was developed by AHRQ, should minimize the degree to which administrative coding differences misclassify hospitalizations. Second, our analysis was limited to the study of a single state, and temporal patterns identified in New York hospital utilization may not be applicable to other states with distinct environmental climates and health burdens. Additionally, our dataset consists of only four years’ worth of data, which limits the conclusions that can be drawn especially for conditions with low admission rates or those that exhibit more heterogeneity in their temporal patterns. Admission rates were also published as monthly totals, which precludes analysis of daily or weekly variation. Another potential limitation is the possibility that certain hospitals systematically undercoded or miscoded certain diagnoses. However, inconsistent coding practices would add noise to the data therefore biasing the finding of seasonality in such conditions towards not successfully identifying statistically significant seasonality. We also note that seasonal variation may be driven by time-dependent changes in incidence of disease, time-dependent severity of disease, or other sources such as hospital capacity. Regardless of the underlying etiology of variation though, the identification of a quantifiable pattern of temporal variation in condition-specific hospital utilization suggests the existence of a seasonal factor or factors underlying temporal patterns in health care utilization. Lastly, it is difficult to assert the statistical significance of the seasonality modeled by the STL method for conditions identified to have low amplitude and low seasonal coefficients. Nonetheless, because such a substantial number of conditions did exhibit strong seasonality, the full list of conditions with statistically significant seasonality were considered, including those with weaker patterns. This was done, in part, to suggest the possible merit of repeating a similar analysis on a larger scale including more data elements for the conditions with typically less frequent admissions.

In conclusion, the application of data-mining techniques to hospital utilization data work contributes not only to a broader understanding of seasonal variation amongst conditions that were previously unstudied, but also furthers the establishment of a rigorous quantitative approach and indices describing temporal variation for the investigation of seasonal variation in future studies. We envision that such future research aimed at identifying seasonal factors driving variation in condition-specific hospital utilization may ultimately lead to new strategies to prevent the need for these hospitalizations. Furthermore, information regarding temporal variation in highly prevalent conditions requiring significant hospital resources may guide the allocation of scarce resources regarding the capacity and operations of hospitals and emergency departments.

## Supporting information

S1 Fig Representative raw utilization data for 8 conditions with both large and small seasonal effects. Raw utilization data are displayed for selected CCS conditions including cancer of bronchus and lung (CCS: 19); gout and other crystal arthropathies (CCS: 54); disorders of teeth and jaw (CCS: 136); skull and face fractures (CCS: 228); genitourinary symptoms and ill-defined conditions (CCS: 163); peripheral and visceral arteriosclerosis (CCS: 114); skin and subcutaneous tissue infection (CCS: 197); and epilepsy convulsions (CCS: 83) with seasonal coefficient (Sc).
(TIF)

S2 Fig Summary of inclusion and exclusion criteria of CCS condition category codes for temporal variation analysis and clustering. Codes were excluded due to missing data or lack of pertinence to medical conditions, which included codes that referred to both non-disease and administrative groupings. Remaining conditions were included in seasonality analysis.
(TIFF)

S1 Table Clustering results for k-means (n = 3) clustering of conditions with summer peaks in hospital utilizations. Clustering number, CCS code, and condition description are shown along with corresponding indices of temporal variation for seasonal coefficient, mean oscillation width, trend coefficient, and trend-linearity.
(XLSX)

## Abbreviations

AHRQ: Agency for Healthcare Research and Quality
CCS: Clinical Classification Software
SEDD: State Emergency Department Databases
SID: State Inpatient Databases
STL: Seasonal-Trend Decomposition based on LOESS
