# Supplementary figures and images for: Discovery of temporal and disease association patterns in condition-specific hospital utilization rates
Source: PLoS One. 2017 Mar 29;12(3):e0172049. doi: 10.1371/journal.pone.0172049 (PMC5371293; doi:10.1371/journal.pone.0172049)

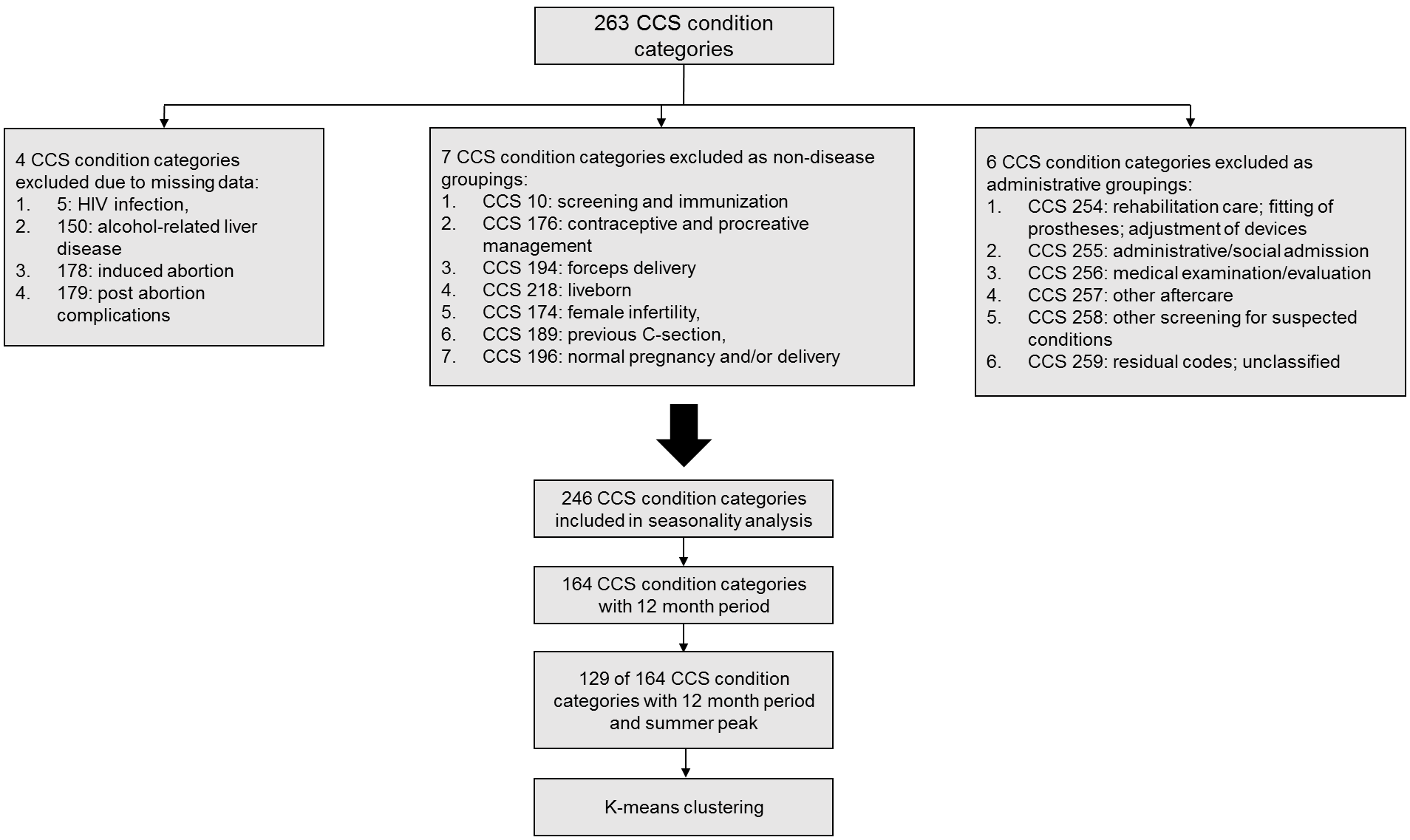

Supplement: S1 Fig — Raw utilization data are displayed for selected CCS conditions including cancer of bronchus and lung (CCS: 19); gout and other crystal arthropathies (CCS: 54); disorders of teeth and jaw (CCS: 136); skull and face fractures (CCS: 228); genitourinary symptoms and ill-defined conditions (CCS: 163); peripheral and visceral arteriosclerosis (CCS: 114); skin and subcutaneous tissue infection (CCS: 197); and epilepsy convulsions (CCS: 83) with seasonal coefficient (Sc). (TIF) [file pone.0172049.s001.tif]

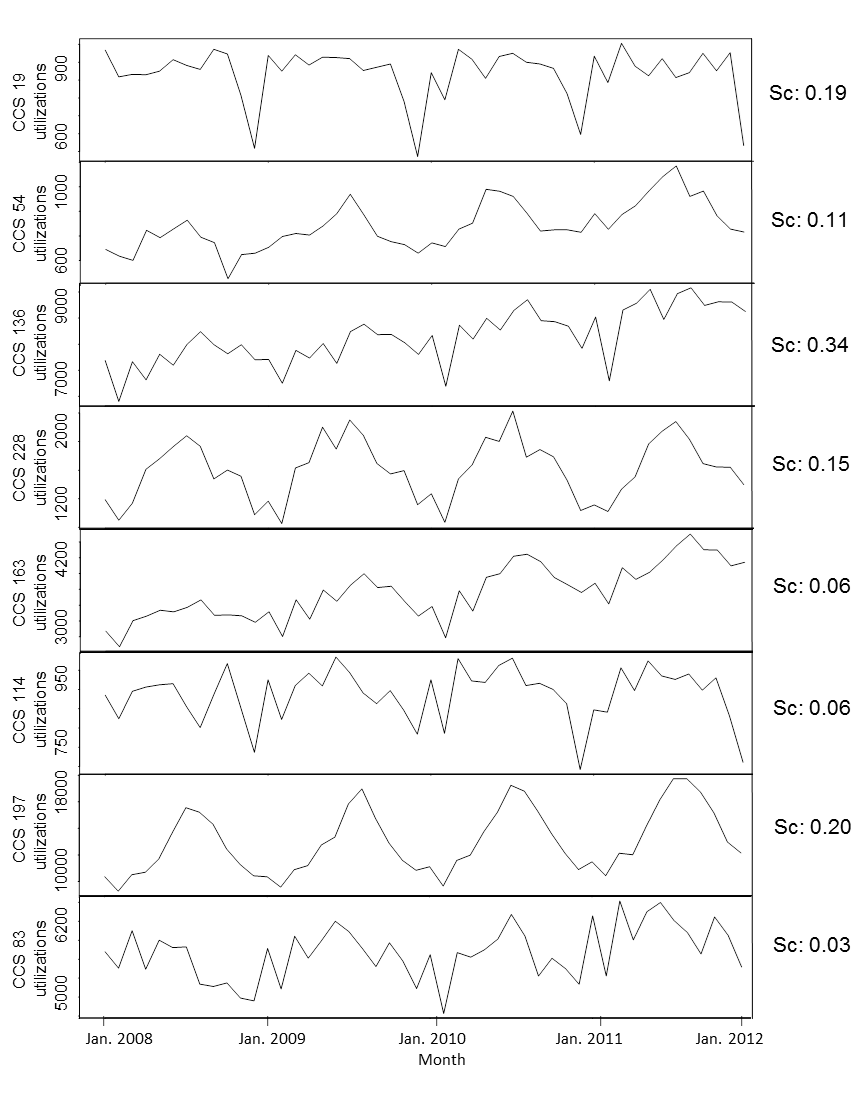

Supplement: S2 Fig — Codes were excluded due to missing data or lack of pertinence to medical conditions, which included codes that referred to both non-disease and administrative groupings. Remaining conditions were included in seasonality analysis. (TIFF) [file pone.0172049.s002.tiff]
